# Rationally Designed Bimetallic Co–Ni Sulfide Microspheres as High-Performance Battery-Type Electrode for Hybrid Supercapacitors

**DOI:** 10.3390/nano12244435

**Published:** 2022-12-13

**Authors:** John Anthuvan Rajesh, Jong-Young Park, Ramu Manikandan, Kwang-Soon Ahn

**Affiliations:** 1 School of Chemical Engineering, Yeungnam University, Gyeongsan 712-749, Republic of Korea; 2 Department of Energy and Materials Engineering, Dongguk University, Seoul 04620, Republic of Korea

**Keywords:** transition metal sulfides, microspheres-like structures, battery-type supercapacitor, hybrid supercapacitor, energy density

## Abstract

Rational designing of electrode materials is of great interest for improving the performance of battery-type supercapacitors. The bimetallic NiCo_2_S_4_ (NCS) and CoNi_2_S_4_ (CNS) electrode materials have received much attention for supercapacitors due to their rich electrochemical characteristics. However, the comparative electrochemical performances of NCS and CNS electrodes were never studied for supercapacitor application. In this work, microsphere-like bimetallic NCS and CNS structures were synthesized via a facile one-step hydrothermal method by controlling the molar ratio of Ni and Co precursors. The physico-chemical results confirmed that microsphere-like structures with cubic spinel-type NCS and CNS materials were successfully fabricated by this method. When tested as the supercapacitor electrode materials, both NCS and CNS electrodes exhibited battery-type behavior in a three-electrode configuration with outstanding electrochemical performances such as specific capacity, rate performance and cycle stability. Impressively, the CNS electrode delivered a high specific capacity of 430.1 C g^−1^ at 1 A g^−1^, which is higher than 345.9 C g^−1^ of the NCS electrode. Furthermore, the NCS and CNS electrodes showed a decent cycling stability with 75.70 and 84.70% capacity retention after 10,000 cycles. Benefiting from the electrochemical advantage of CNS microspheres, we fabricated a hybrid supercapacitor (HSC) device based on CNS microspheres (positive electrode) and activated carbon (AC, negative electrode), which is named as CNS//AC. The assembled CNS//AC HSC device showed a large energy density of 41.98 Wh kg^−1^ at a power density of 800.04 W kg^−1^ and displayed a remarkable cycling stability with a capacity retention of 91.79% after 15,000 cycles. These excellent electrochemical performances demonstrate that both bimetallic NCS and CNS microspheres may provide potential electrode materials for high performance battery-type supercapacitors.

## 1. Introduction

Recently, the battery-type supercapacitor draws much interest due to its fast charge/discharge capability, high theoretical capacity, high-power density, and long-term cycle stability [1,2,3]. Battery-type supercapacitor electrode materials store energy via a Faradaic redox reaction, which involves a diffusion-controlled charge transfer between the electrode and electrolyte interface [4,5]. Battery-type electrode materials with improved electrochemical characteristics such as richer Faradaic reactions, different valence states, and low resistivity are required to attain a high specific capacity and high power/energy densities of a hybrid supercapacitor (HSC) device [1,2,3,4,5,6,7,8]. In addition to that, rational designing of the composition-controlled electrode materials is crucial to achieve a high power and energy density of the HSC device [2,3,6,7,8,9].

Among the various battery-type materials, metal sulfides are favorable energy storage materials due to their high electrochemical properties such as intrinsic electronic conductivity, favorable redox properties, low electronegativity, and lower resistance [9,10,11]. Compared to single metal sulfides, bimetallic sulfides exhibit variable oxidation states, high electrical conductivity, and high redox properties [12,13,14]. Especially, NiCo_2_S_4_ (NCS) and CoNi_2_S_4_ (CNS) are considered as the promising electrode materials for battery-type supercapacitors because of higher electronic conductivity, high theoretical capacity, and richer redox reaction chemistry [15,16,17,18,19]. The synthesis of bimetallic NCS and CNS electrode materials with controllable stoichiometry composition were elaborately studied and most of the synthesis methods reported elsewhere are hydrothermal or solvothermal [20,21,22,23,24,25]. Additionally, various morphologies of NCS and CNS have been synthesized successfully such as mushroom-like [26], burl-like [27], nanotube arrays [15,28], hollow spheres [29], nanoparticles [18,25], flaky arrays [24], 3D-flower-like [23], hollow cube [19], nanosheet arrays [21], and nanoneedles [20]. Moreover, as-prepared different NCS and CNS morphologies showed excellent electrochemical supercapacitor properties in a three-electrode configuration [15,18,19,20,21,22,23,24,25,26,27,28,29]. For example, Pengfei et al. reported the synthesis of NiCo_2_S_4_ hollow cages, which displays a high specific capacitance of 1382 F g^−1^ at 1 A g^−1^ [30]. Recently, Souleymen et al. prepared ultrathin CoNi_2_S_4_ nanosheets and this battery-type electrode exhibits a specific capacitance of 247 mAh g^−1^ at 8 A g^−1^ [31]. Wang et al. have synthesized carbon-NiCo_2_S_4_ hetero-structured nanosheet arrays by two-step method (hydrothermal and CVD). This carbon-NiCo_2_S_4_ electrode shows ultrahigh specific capacitance of 1893.2 F g^−1^ [32]. Three-dimensional flower-like CoNi_2_S_4_ was prepared by Lemu et al. and exhibits a high areal capacitance of 6.528 F cm^−2^ at a current density of 6 mA cm^−2^ [23]. These previous findings indicate that the bimetallic NCS and CNS materials have large potential in battery-type supercapacitor application due to their improved electrochemical properties.

Benefiting from the bimetallic NCS and CNS electrode materials, this work has been concentrated to determine the effect of Ni/Co metals stoichiometry (1:2 and 2:1) on the electrochemical performances of NCS and CNS materials for battery-type supercapacitor application. We have successfully prepared the microspheres-like bimetallic NCS and CNS electrode materials by a facile one-step hydrothermal method. A comparative electrochemical supercapacitor studies were carried out for the first time. The supercapacitor properties of as-synthesized NCS and CNS electrodes revealed a good specific capacity, reasonable rate capability, and excellent cyclic stability in a three-electrode system. Specifically, the microspheres-like CNS electrode exhibited a maximum specific capacity (430.1 C g^−1^ at 1 A g^−1^) than NiCo_2_S_4_ electrode (345.9 C g^−1^ at 1 A g^−1^). In addition, an HSC device fabricated by CNS microspheres as the positive electrode and activated carbon (AC) as the negative electrode, labeled as CNS//AC. The CNS//AC HSC device delivered a high specific energy (41.98 Wh kg^−1^) and specific power (800.04 W kg^−1^) with excellent cyclic stability (91.79% capacitance retention after 15,000 charge/discharge cycles).

## 2. Experimental

Analytical grade chemicals such as nickel nitrate hexahydrate (Ni(NO_3_)_2_·6H_2_O), cobalt nitrate hexahydrate (Co(NO_3_)_2_·6H_2_O), thioacetamide (C_2_H_5_NS), potassium hydroxide (KOH), AC, acetylene black, poly(vinylidene fluoride) (PVDF), N-methyl-2-pyrrolidone, and ethylene glycol (EG, HOCH_2_CH_2_OH) were procured from Sigma Aldrich, South Korea. Ethanol (C_2_H_5_OH) was purchased from DUKSAN chemicals, South Korea. Deionized water (DI H_2_O) was used throughout the experiments. Commercial nickel foam (Ni-foam) was purchased from MTI, South Korea.

Microspheres-like NCS and CNS samples were synthesized via a facile one-step hydrothermal method. In a typical synthesis method of NCS microspheres, 5 mM Ni(NO_3_)_2_.6H_2_O, 10 mM Co(NO_3_)_2_.6H_2_O, and 12 mM thioacetamide were dissolved in 40 mL EG solvent by continuous stirring to obtain a homogeneous solution. Next, the as-prepared light pink color solution was transferred into 50 mL capacity autoclave. The autoclave was closed tightly, and the hydrothermal treatment was carried out in a muffle furnace (Wisd Laboratory Instruments, South Korea) at 180 °C for 12 h. After the reaction completion, the black color precipitates were collected and thoroughly washed with DI H_2_O and C_2_H_5_OH, and then dried in an oven at 70 °C for overnight. The CNS microspheres were obtained by the same synthesis process with 5 mM Co(NO_3_)_2_.6H_2_O, 10 mM Ni(NO_3_)_2_.6H_2_O, and 12 mM thioacetamide. The materials characterization and electrochemical studies were given in Supplementary Materials.

## 3. Results and Discussion

**Figure 1:**
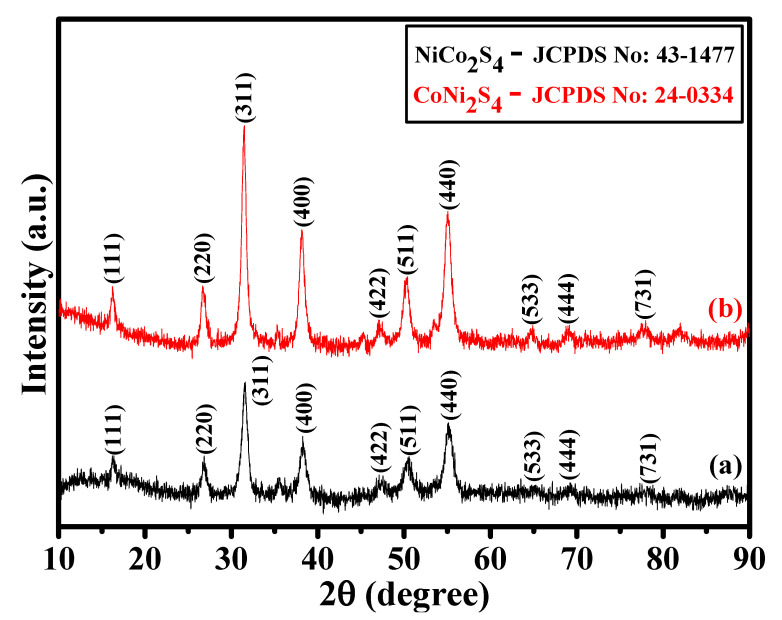
(**a**,**b**) XRD patterns of the NCS and CNS samples, respectively.

The crystalline phases of the as-prepared NCS and CNS samples were identified by X-ray diffraction (XRD). As shown in Figure 1a,b, the crystalline peaks suggest that both the samples were mainly composed of cubic spinel structures. All the characteristic peaks identified at 16.3°, 26.8°, 31.6°, 38.3°, 47.4°, 50.5°, 55.3°, 65.1°, 69.3°, and 77.91° can be respectively indexed into (111), (220), (311), (400), (422), (511), (440), (533), (444), and (731) planes of the cubic spinel NiCo_2_S_4_ (JCPDS No: 43-1477) and CoNi_2_S_4_ (JCPDS No: 24-0334) [17,18,24,33]. It is very difficult to differentiate the NiCo_2_S_4_ and CoNi_2_S_4_ crystalline phases due to their similar peak position. To better understand the difference between NiCo_2_S_4_ and CoNi_2_S_4_ phases, we verified the space group and a primitive unit cell value. Both NiCo_2_S_4_ and CoNi_2_S_4_ samples were belonging to the Fd-3m space group. However, the primitive unit cell values of NiCo_2_S_4_ and CoNi_2_S_4_ were 9.4177 Å and 9.4279 Å, respectively [24,25]. A small difference in the primitive unit cell values clearly distinguish the crystalline phases of NiCo_2_S_4_ and CoNi_2_S_4_ in this work. In addition, no other peaks found in the XRD patterns, suggesting the formation of pure cubic spinel NiCo_2_S_4_ and CoNi_2_S_4_ phases. Moreover, the XRD results suggest that good crystalline NiCo_2_S_4_ and CoNi_2_S_4_ phases were successfully prepared by our one-step hydrothermal method.

**Figure 2:**
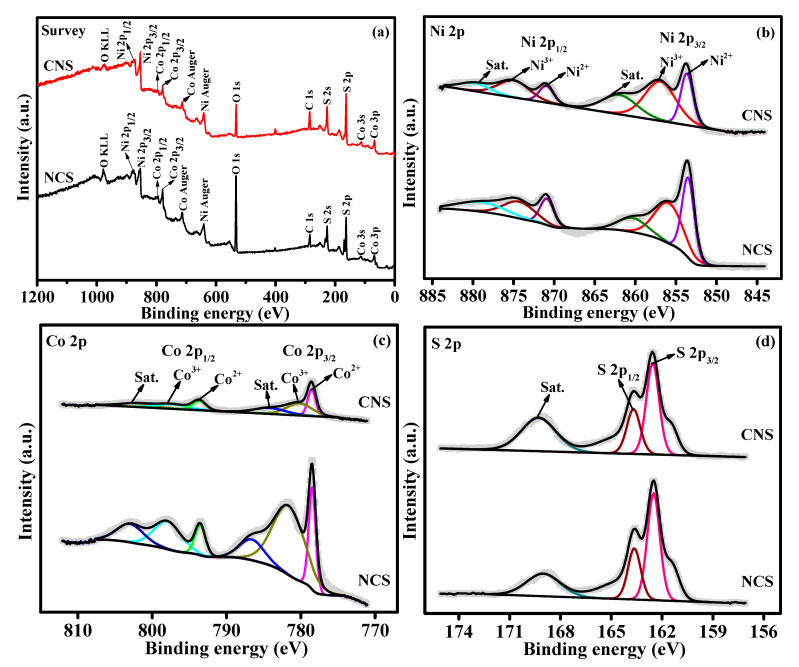
(**a**) The XPS survey scan spectrum of NCS and CNS samples. The deconvoluted spectra of (**b**) Ni 2p, (**c**) Co 2p, and (**d**) S 2p.

The elemental oxidation states and surface chemical composition of as-synthesized NCS and CNS materials were evaluated by X-ray photoelectron spectroscopy (XPS). The survey scan spectrum of NCS and CNS samples (Figure 2a) indicates the presence of Ni, Co, and S peaks. The Ni 2p spectrum (Figure 2b) consists of two spin-orbit doublets (Ni^2+^ and Ni^3+^) and two shakeup satellites (identified as “sat.”). The two main peaks at 853.6 and 871.2 eV were ascribed to Ni^2+^, while other two best fitted peaks at 856.8 and 874.7 eV were indexed to Ni^3+^ [34,35,36,37,38]. The deconvoluted Co 2p spectrum also (Figure 2c) shows two spin−orbit doublets, which are characteristic peaks of Co(II) and Co(III). Specifically, the fitted peaks at 778.5 and 793.5 eV were related to Co(II) ions. The remaining two fitted peaks at 781.9 and 798.2 eV were attributed to Co(III) ions. Furthermore, two satellite peaks were also identified at 855.7 and 873.1 eV [34,35,36,37,38]. As shown in Figure 2d, the S 2p spectrum can be deconvoluted into two main peaks located at 162.5 and 163.6 eV, which correspond to S 2p3/2 and S 2p1/2 of metal-sulfur bonds in the NCS and CNS samples, respectively [34,35,36,37,38]. The other peak at 170.7 eV due to the shakeup satellite peak [24,33,34]. The XPS results demonstrate that NCS and CNS samples have a mixed composition, containing Ni^2+^, Ni^3+^, Co^2+^, Co^3+^ and S^2−^, which are in good accordance with the results reported for NiCo_2_S_4_ and CoNi_2_S_4_ in the literature [15,19,24,33].

**Figure 3:**
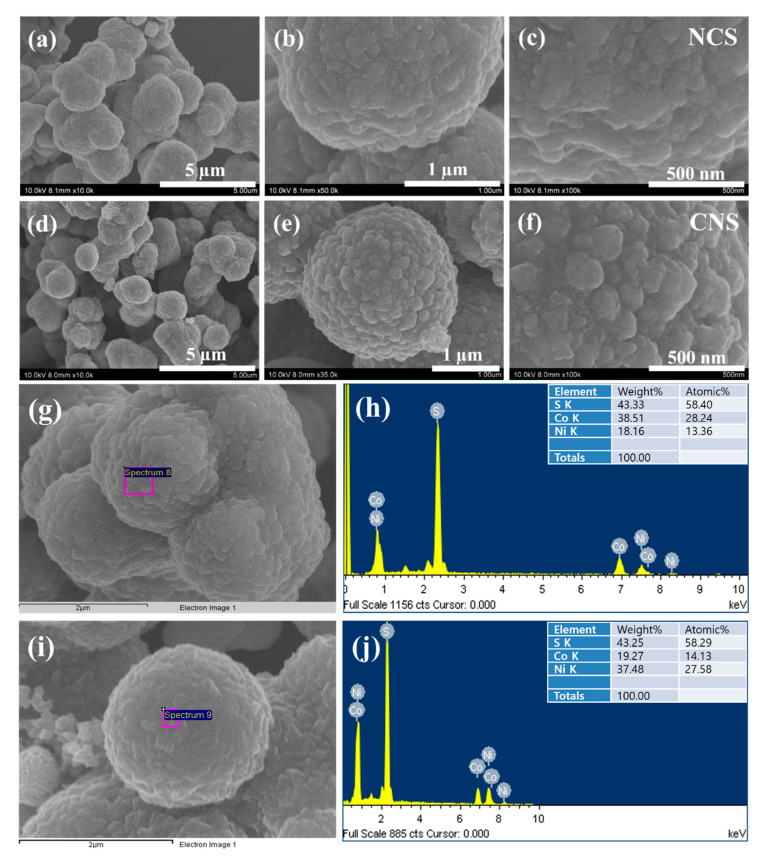
FE-SEM images of (**a**–**c**) NCS microspheres and (**d**–**f**) CNS microspheres. (**g**,**i**) Electron images of NCS and CNS samples, respectively. (**h**,**j**) EDX spectra of NCS and CNS, respectively.

The morphological, structural features, and elemental composition of the as-synthesized NCS and CNS samples were studied by field-emission scanning electron microscopy (FE-SEM), field-emission transmission electron microscopy (FE-TEM), high resolution TEM (HRTEM), selected-area electron diffraction (SAED), and energy dispersive X-ray analysis (EDX). Figure 3 shows the typical FE-SEM images of NCS and CNS samples synthesized by a simple one-step hydrothermal method. Even though, the molar ratio of Ni and Co metals was different in NCS and CNS samples, the FE-SEM images in Figure 3a–f expose that both sulfide materials were quite similar in morphology. The low-magnification FE-SEM (Figure 3a,d) images show that the NCS and CNS samples consist of many sphere-like structures with an average diameter of 2 μm. The NCS and CNS microspheres surface was very rough and comprised of many nanoparticles, which can be observed from high magnified FE-SEM images (Figure 3b,e). Figure 3c,f clearly distinguish the morphology difference between NCS and CNS samples. The surface of the NCS sample was flat and rough, while the CNS sample surface was made up of roughed nanoparticles. It can be clearly noted that the nanoparticles attached to each other and formed well-defined microsphere-like structures in CNS compared with that of NCS sample. The elemental composition of the NCS and CNS samples is further analyzed by FE-SEM-EDX. Figure 3g,i show the electron microscopy images of NCS and CNS samples, respectively. The corresponding EDX patterns in Figure 3h,j indicates that the as-synthesized microspheres were mainly comprised of Ni, Co, and S without any other impurity elements. The calculated atomic percentages of Ni, Co and S were in the ratio of 13.36:28.24:58.40 and the atomic percentages of Co, Ni and S were in the ratio of 14.13:27.58:58.29, which are close to the theoretical values of NiCo_2_S_4_ and CoNi_2_S_4_ (1:2:4).

**Figure 4:**
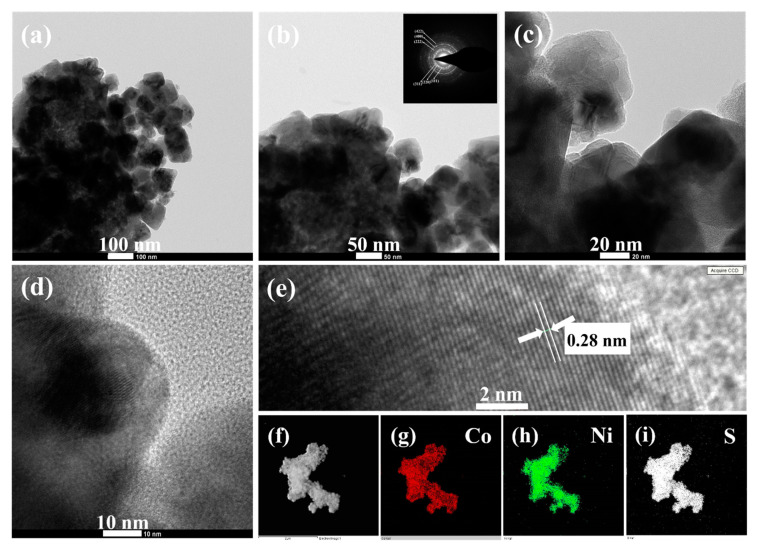
(**a**–**c**) TEM images (**d**) high magnified TEM image, (**e**) HRTEM image of CNS microsphere-like structure, and (**f**–**i**) HAADF-STEM elemental mapping images of CNS sample. The inset in (**b**) is the SAED pattern.

The detailed morphological and crystalline structures of the CNS sample were further analyzed by TEM, HRTEM, and SAED. Typical low magnification TEM image in Figure 4a apparently shows the microsphere-like morphology of CNS sample, which is consistent with the SEM observation (Figure 3e). Notably, the magnified TEM result (Figure 4b) clearly illustrates that as-prepared CNS sample composed of several nanoparticles, which are intact each other to develop microsphere-like morphology. The corresponding SAED pattern (Inset Figure 4b) displays well-defined rings, which can be indexed to the (111), (220), (311), (222), (400), and (422) planes of CoNi_2_S_4_ phase, further confirms that the polycrystalline nature of CoNi_2_S_4_ sample [21,37]. The interconnected nanoparticle’s structure was further characterized by high magnified TEM (Figure 4c,d). These nanoparticles boundaries were clear, and no amorphous layers were found on the high magnified TEM images, indicating the high crystallinity of as-prepared microspheres-like CNS sample. Furthermore, a well resolved lattice fringes can be observed on the atomic scale (Figure 4e) and the calculated interplanar spacing is about 0.28 nm corresponding to the (311) lattice spacing of cubic spinel CoNi_2_S_4_ [26,31,37]. The elemental mapping of the CNS sample was further analyzed by high angle annular dark field (HAADF) scanning TEM (STEM). Figure 4f–i shows the STEM-HAADF image and corresponding elemental maps of CNS sample. The results clearly proved that Co, Ni and S elements were uniformly distributed within the CNS sample, which further confirms the formation phase pure CoNi_2_S_4_.

**Figure 5:**
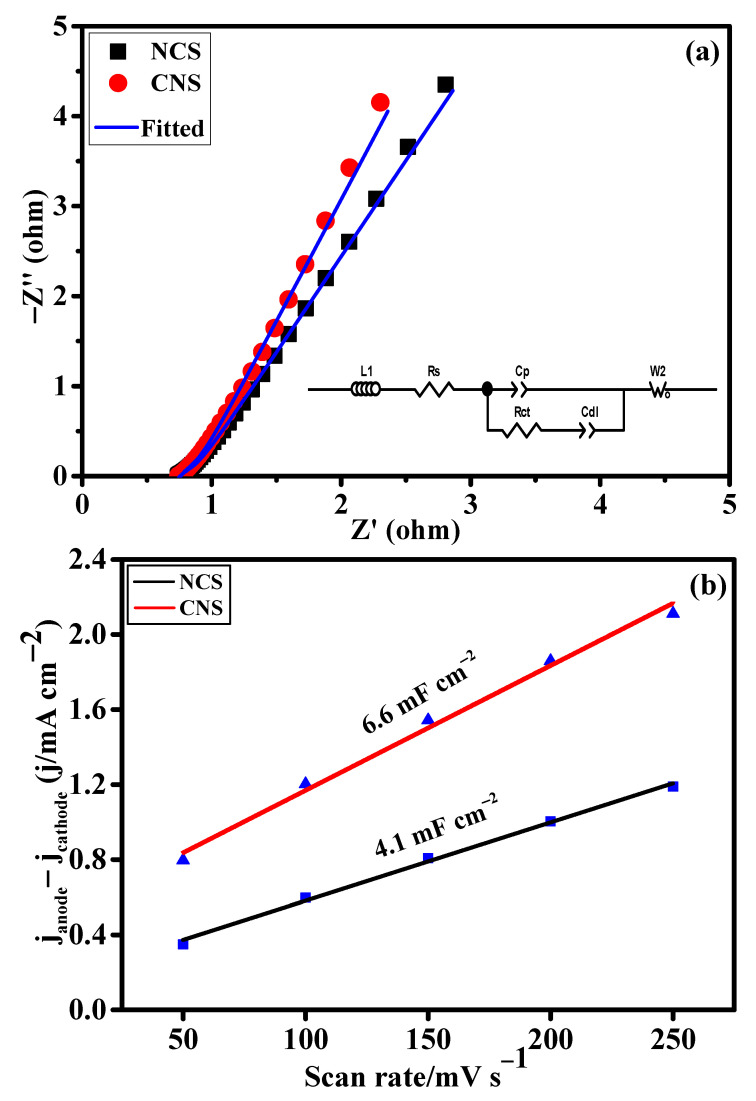
The intrinsic electrochemical properties of the as-prepared NCS and CNS electrodes. (**a**) Nyquist plots and the inset is equivalent circuit model and (**b**) plots of the current density differences vs. the scan rate.

The intrinsic electrochemical properties of the as-prepared NCS and CNS electrode materials were evaluated using the electrochemical impedance spectroscopy (EIS) and electrochemical double-layer capacitance (C_dl_) analyses. Figure 5a shows the EIS spectra of NCS and CNS electrodes. The Nyquist plots were fitted using the equivalent circuit model in the inset Figure 5a and the resulting data were displayed in Table S1. Obviously, CNS electrode delivers the low charge-resistance (0.72 Ω) and charge-transfer resistance (0.27 Ω) than NCS electrode, indicating the high conductivity and efficient redox kinetics of the CNS electrode. To evaluate the electrochemical surface area of the NCS and CNS electrodes, the C_dl_ values were calculated by assessing the CV curves in the non-Faradaic region at various scan rates from 50 to 250 mV s^−1^ (Figure S1). It is well known that the C_dl_ values were directly related to the electrochemical surface areas of the corresponding electrode materials. Figure 5b shows the plot of current density as a function of scan rates. From the plot, the C_dl_ values of the NCS and CNS electrodes were determined to be 4.1 and 6.6 mF cm^−2^, respectively (Figure 5b). This result indicates that CNS electrode had high number of electroactive sites for the facile transport of electrolyte ions to the electrode surface than the NCS electrode.

**Figure 6:**
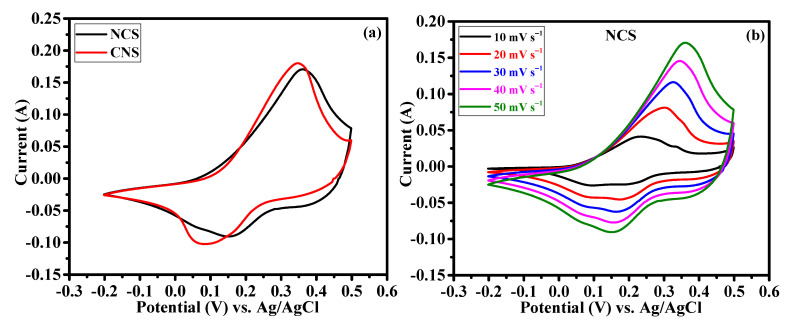

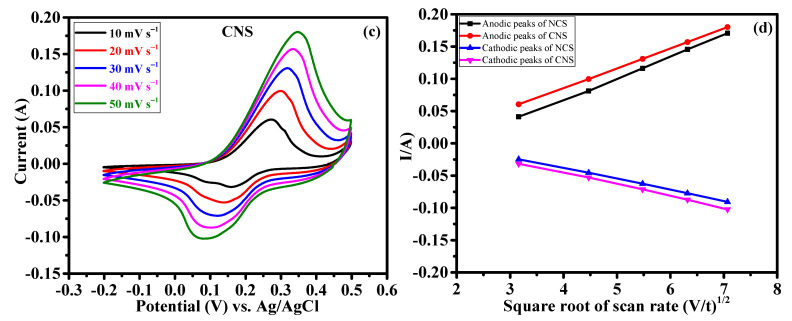
Cyclic voltammetry performance of NCS and CNS electrodes. (**a**) comparative CV curves, (**b**,**c**) detailed CV curves of NCS and CNS electrodes at different sweep rates, respectively. (**d**) The relationship between the cathodic/anodic peak current and the square root of the scan rate for NCS and CNS electrodes.

The effect of Ni/Co metal ratio (1:2 and 2:1) on the electrochemical supercapacitor performances were investigated by cyclic voltammetry (CV), galvanostatic charge-discharge (GCD) and continuous charge/discharge stability studies. Figure 6a reveals the comparative cyclic voltammograms of the NCS and CNS electrodes at the same sweep rate of 50 mV s^−1^ within the potential window of –0.2~0.5 V. Clearly, both the CV curves exhibit redox peaks, indicating the intercalation and deintercalation of OH^–^ ions follow the Faradaic reaction. The broad and strong redox peaks further confirming the typical battery-type behavior of NCS and CNS electrodes [3,4,5,6,7,8]. Moreover, the closed CV areas were different for the NCS and CNS electrodes, indicating that both electrodes have different charge storage capacity. The CV area of the CNS electrode is slightly higher than that of NCS electrode, demonstrating that the well-deserved microsphere-like CNS sample has higher capacity performance. The distinct redox peaks of the NCS and CNS electrodes can be associated with the reversible Faradaic reactions of Co^2+^/Co^3+^/Co^4+^ and Ni^2+^/Ni^3+^ [31,33,35,37]. Figure 6b and c shows the CV curves of NCS and CNS electrodes recorded at a various sweep rates of 10, 20, 30, 40 and 50 mV s^−1^, respectively. As the scan rate increases from 10 to 50 mV s^−1^, the CV areas and the anodic/cathodic peak currents were increased, indicating the excellent reversible Faradaic behavior of these electrodes. In addition, the redox peak shift also found with the increasing scan rate, which can be attributed to the polarization and internal resistance of the electrodes [24,25,34,37]. Figure 6d shows the plot of square root of the scan rate Vs. peak current. The plot clearly reveals a linear dependence of peak currents with sweep rate, indicating the NCS and CNS electrodes are highly reversible, and the redox reactions were diffusion-controlled process [39,40,41].

**Figure 7:**
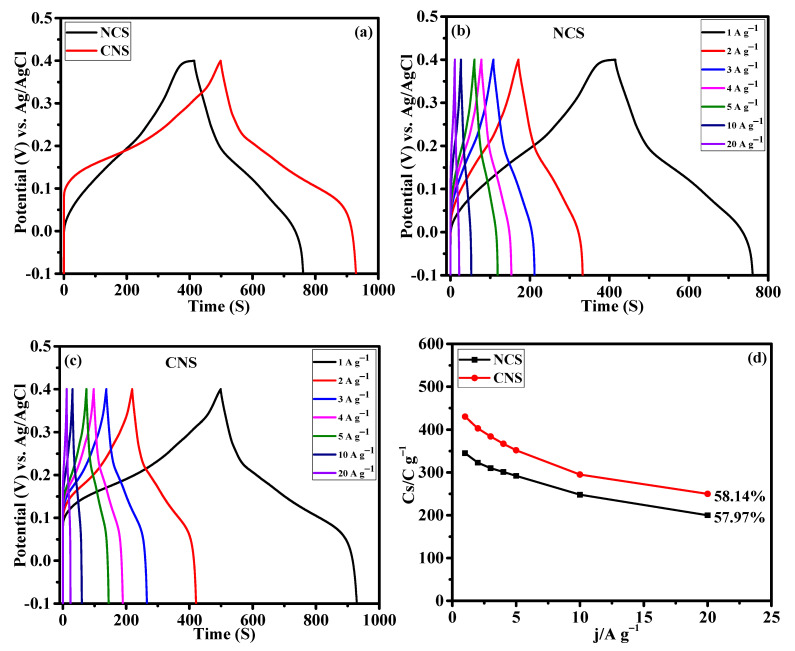
Galvanostatic charge discharge performance of NCS and CNS electrodes. (**a**) comparative GCD profile, (**b**,**c**) detailed GCD curves of NCS and CNS electrodes at different current densities, respectively. (**d**) The rate performance of NCS and CNS electrodes.

The capacity performance of the NCS and CNS electrodes were estimated by GCD measurement. Figure 7a present the GCD profile of NCS and CNS electrodes measured at the same current density of 1 A g^−1^. The charge and discharge curves exhibit the clear plateaus within the potential window of –0.1~0.4 V, further verifying the battery-type characteristic of these electrodes, which is consistent with the CV results (Figure 6a–c). The NCS electrode shows lower discharge time compared to CNS electrode. The specific capacity values of the electrodes were calculated from the discharge curves according to the Equation (S1). The calculated specific capacity values of NCS and CNS electrodes were 345.9 and 430.1 C g^−1^ at 1 A g^−1^, respectively. Table S2 compares the electrochemical performance of the NCS and CNS electrode materials with sulfide-based electrode materials. From Table S2, it is clear that the performances of NCS and CNS electrode were comparable to the reported sulfide-based electrode materials for supercapacitor application. Compared to other morphologies, the unique microspheres-like NCS and CNS electrodes were beneficial for supercapacitor application. The CV integral area (Figure 6a) and specific capacity value of the CNS electrode is larger than that of NCS electrode, which is possibly due to the higher Ni concentration of CNS electrode [42,43]. Chen et al. have demonstrated the effect of Ni^2+^ concentration on the electrochemical performance of Ni-Co-S nanosheets [42]. In our previous work, we demonstrated the effect of Ni^2+^ and Co^2+^ concentration on the battery-type supercapacitor performance of NiCo_2_Se_4_ and CoNi_2_Se_4_ materials [42]. From the previous studies, it is found that the cobalt sulfides show higher redox potentials than nickel sulfides due to their inherent electrochemical response [44,45,46]. Consequently, the Ni-rich CoNi_2_S_4_ microspheres exhibited improved electrochemical properties than NiCo_2_S_4_ microspheres. The detailed GCD profiles of the NCS and CNS electrodes at diverse current densities of 1, 2, 3, 4, 5, 10 and 20 A g^−1^ (Figure 7b,c), exhibit symmetrical GCD curves at all the tested current densities, suggesting highly reversible behavior of oxidation and reduction reactions. The CNS electrode delivered the specific capacity values of 430.1, 403.8, 384.9, 367.6, 352.5, 295.0, and 250.0 C g^−1^ at the current densities of 1, 2, 3, 4, 5, 10, and 20 A g^−1^, respectively. Based on the discharge curves of NCS electrode, the calculated specific capacity values were 345.9, 323.4, 310.8, 301.2, 292.5, 259.0, and 200.0 C g^−1^ at 1, 2, 3, 4, 5, 10, and 20 A g^−1^, respectively. Rate capability is one of the key factors to analyze the supercapacitor performance. The rate capability curves (specific capacity values Vs. the applied current density) of NCS and CNS electrodes were plotted in Figure 7d. Obviously, the two samples delivered a reasonable specific capacity retention of 57.82% and 58.14% for NCS and CNS electrodes, respectively. This result indicates that the NCS and CNS electrodes possess outstanding rate capability because of mass OH^−^ ions diffusion at the electrode–electrolyte interface [39,40,41].

**Figure 8:**
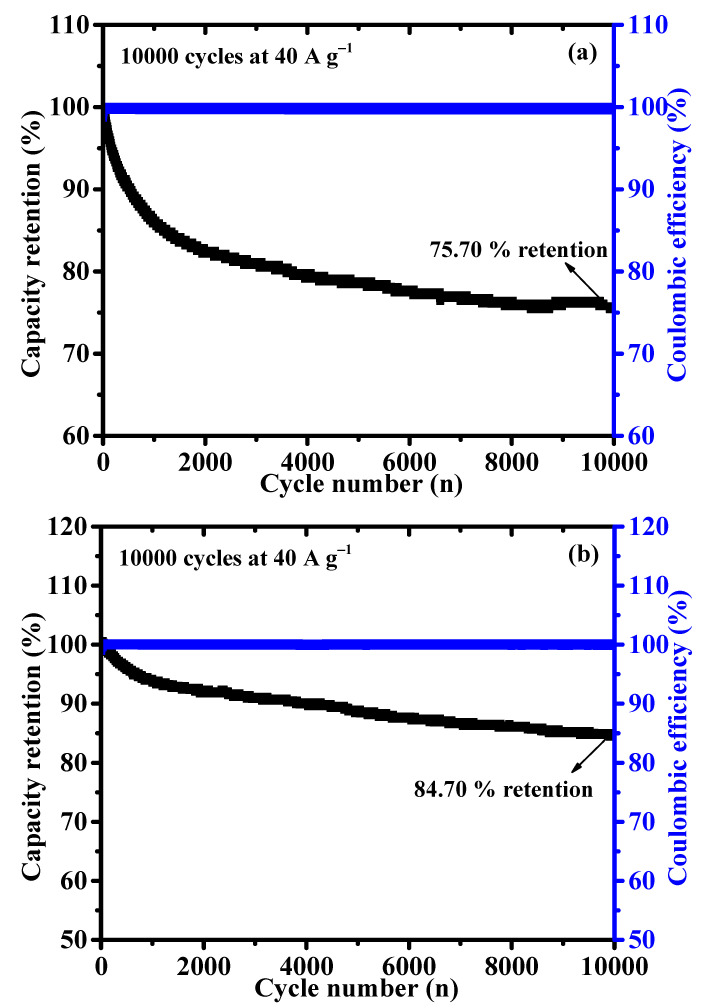
(**a**,**b**) Long–term cyclability of the NCS and CNS electrode at 40 A g^−1^ for 10,000 cycles, respectively.

The long-term cyclic stability is another essential feature for battery-type supercapacitor application. The cycling stability of NCS and CNS samples were assessed by continuous charge/discharge test over 10,000 cycles at a constant current density of 40 A g^−1^. As shown in Figure 8, the capacity retention of NCS and CNS electrodes remained 75.70 and 84.70% after 10,000 cycles, demonstrating excellent cyclic stability of both sulfide-based electrodes. As expected, CNS electrode shows a good cyclic stability than the NCS electrode, which is good agreement with the CV and GCD results. The stable cycling performance of the NCS and CNS electrodes can be related to the unique microspheres-like morphology and their high reversible redox reactions. Furthermore, the Coulombic efficiency (η) of the NCS and CNS electrodes was calculated from the charge and discharge times using the Equation (S2). The Coulombic efficiency of the NCS and CNS electrodes was 100% over the 10,000 cycles (blue line in Figure 8), demonstrating the remarkable reversibility of the Faradaic reactions. To further examine the crystalline phase and morphology changes of CNS electrode after stability study, the XRD and FE-SEM analyses were carried out. As shown in Figure S2, the crystalline phase and surface morphology of the CNS electrode can be well preserved after 10,000 cycles, which further confirms the long-term durability of this CNS electrode material. The superior electrochemical performances of microspheres-like CNS electrode can be attributed to the following characteristics: (1) The as-prepared microspheres-like CNS electrode possesses high electrochemical surface area, which can offer numerous electroactive sites for the intercalation and deintercalation of OH^−^ ions. (2) The microspheres-like morphology having interconnected CNS primary nanoparticles, which possibly increases the efficient pathways for bulk diffusion of electrolyte ions. (3) The lower internal resistance of the CNS electrode can reduce the ion diffusion path, leading to the fast electron transfer between the electrode and electrolyte interface. (4) The microspheres-like morphology can prevent the volume changes during the long-term stability due to the highly interconnected CNS nanoparticles morphology.

Inspired by the enhanced electrochemical properties of microspheres-like CNS electrode, an HSC device was further assembled by utilizing CNS and AC as the positive and negative electrode, respectively. Prior to studying the HSC device, the electrochemical properties of AC electrode were evaluated in a three-electrode system. Figure S3 shows the CV and GCD profiles of AC analyzed using the 3M KOH electrolyte. The shape of the CV curves at various scan rates from 10 to 50 mV s^−1^ was rectangular, indicating electrical double-layer capacitance (EDLC) property [30,31]. In addition, negative electrode works stable within the voltage of −1~0 V. The GCD curves show symmetrical profile, signifying good capacitive behavior of the EDLC. The specific capacitance values of the negative electrode were calculated according to the Equation (S3), and the specific capacitance values were 178.8, 149.6, 95.5, and 42.0 F g^−1^ at 1, 2, 5, and 10 A g^−1^, respectively.

**Figure 9:**
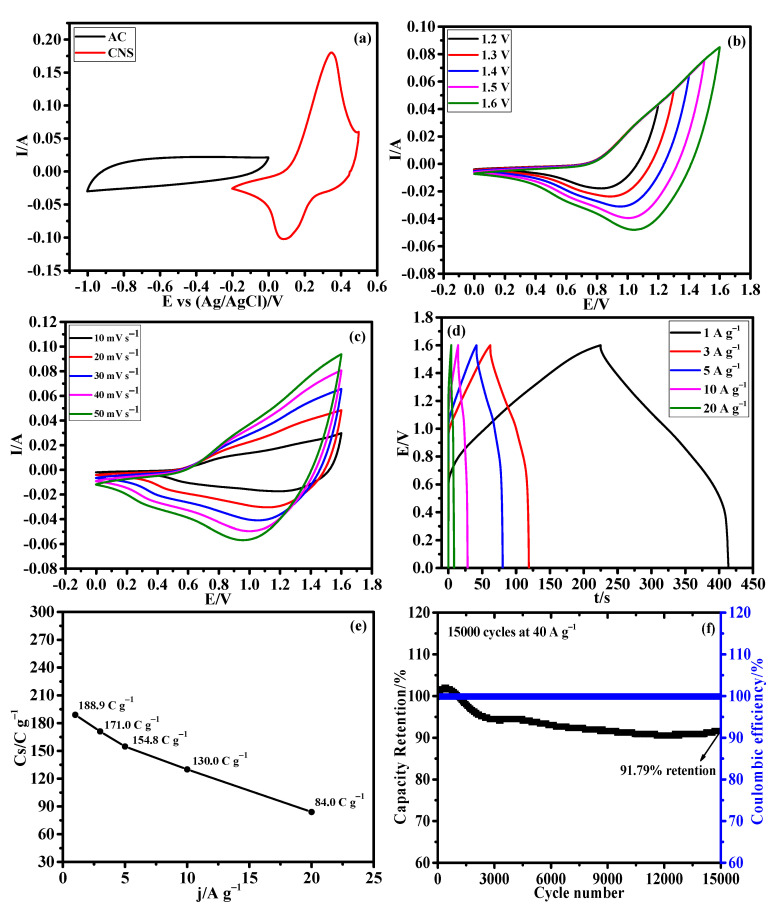
Hybrid supercapacitor performance of CNS//AC device. (**a**) CV curves of AC and CNS electrodes at 50 mV s^−1^, (**b**) CV curves at different potential windows from 0−1.2 to 0−1.6 V, (**c**) CV curves at different scan rates from 10 to 50 mV s^−1^, (**d**) GCD curves at different current densities, (**e**) plot of specific capacities Vs. different current densities, and (**f**) long-term stability performance of the HSC device for 15,000 cycles at 40 A g^−1^.

Afterward, an HSC device was constructed using CNS as the positive electrode, AC as the negative electrode, and 3M KOH aqueous solution as the electrolyte to evaluate the CNS electrode for real time application. The mass ratio of CNS and AC electrodes was calculated using the traditional charge balance equation [47]. Based on the specific capacitance values of positive and negative electrodes, the mass ratio between the CNS and AC was calculated to be 0.15. The assembled HSC device was named as CNS//AC. Figure 9a illustrates the CV curves of the CNS and AC electrodes measured in a three-electrode system at a same sweep rate of 50 mV s^−1^. The EDLC type AC electrode operated from −1 to 0 V and the battery-type CNS electrode works within the potential window of −0.2~0.5 V. Figure 9b shows the CV curves of CNS//AC device at various voltage windows (from 0−1.0 to 0−1.6 V) under a same scan rate of 50 mV s^−1^, which clearly indicates that there is no noticeable polarization until the 1.6 V. Figure 9c shows the detailed CV curves of the CNS//AC HSC device collected at various scan rates from 10 to 50 mV s^−1^, which delivered the both battery-type and EDLC-type electrochemical properties. As the scan rate increases, the shape of the CV curves was retained well, indicating the excellent reversibility of the CNS//AC device. Further, Figure 9d demonstrates the GCD curves of CNS//AC device measured at 1, 3, 5, 10, and 20 A g^−1^ within the potential window of 0~1.6 V. The GCD curves with symmetric nature further demonstrate the charge storage process in the CNS//AC HSC device is highly reversible [30,47]. Specific capacity values of the HSC device were determined using to the Equation (S1) and the calculated specific capacity values were high as 188.9, 171.0, 154.8, 130.0, and 84.0 C g^−1^ at 1, 3, 5, 10, and 20 A g^−1^, respectively. Remarkably, CNS//AC HSC device shows a high specific capacity of 188.9 C g^−1^ at a current density of 1 A g^−1^, and an excellent capacity retention of 68.82% was attained when the current density increased from 1 to 10 A g^−1^ (Figure 9e). The cycling stability (Figure 9f) of the CNS//AC HSC device was evaluated by continuous charge/discharge test over 15,000 cycles at 40 A g^−1^, and a remarkable specific capacity of 91.79% was retained even after 15,000 cycles and showed a high columbic efficiency of 99.3%.

**Figure 10:**
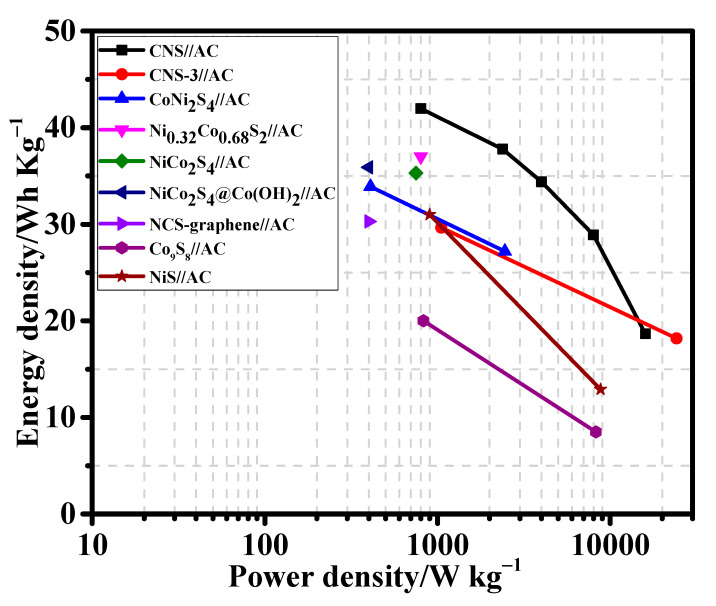
Ragone plot of energy density and power density of CNS//AC HSC device with previously reported supercapacitor devices.

**Figure 11:**
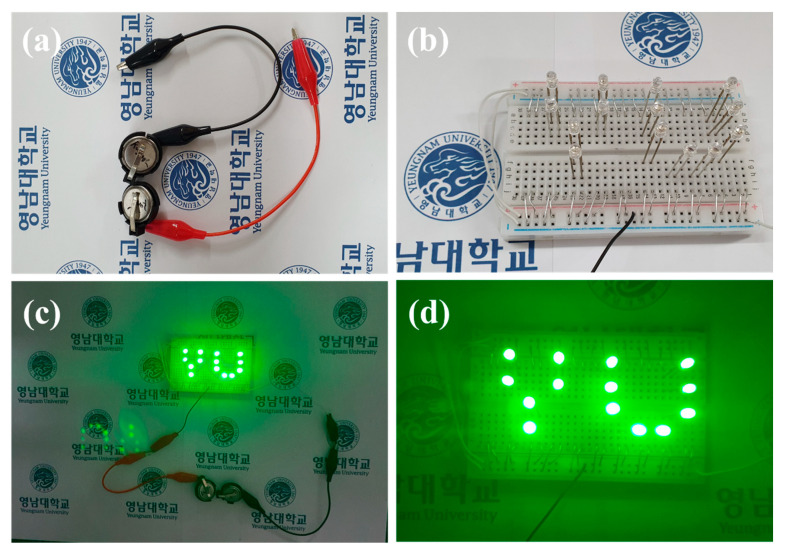
(**a**) Two HSC cells connected in series to light green LEDS, (**b**) YU pattern made up of 14 LEDs, (**c**,**d**) 14 LEDs powered by two serially connected HSC cells.

The energy density and power density values of the CNS//AC HSC device were calculated from the discharge curves (Figure 9d) using the Equations (S4) and (S5). Remarkably, the CNS//AC HSC device delivers a high energy density and power density of 41.98 Wh kg^−1^ and 800.04 W kg^−1^, respectively. The energy densities and power densities of the CNS//AC HSC device, and the reported data for the sulfide-based HSC devices were compared in a Ragone plot, as presented in Figure 10. The energy densities of the CNS//AC HSC device were 41.98, 37.78, 34.4, 28.89, and 18.66 Wh kg^−1^ at the power densities of 800.04, 2386.11, 3200.0, 8000.0, and 15,944.29 W kg^−1^, respectively. These values were greater to those of the previously reported sulfide-based HSC devices such as CNS-3//AC (29.66 Wh kg^−1^ and 1050.55 W kg^−1^) [48], NiCo_2_S_4_ nanotubes//RGO (31.5 Wh kg^−1^ and 156.6 W kg^−1^) [49], CoNi_2_S_4_ nanosheet arrays//AC (33.9 Wh kg^−1^ and 409 W kg^−1^) [21], Ni_1.77_Co_1.23_S_4_//AC (42.7 Wh kg^−1^ and 190 W kg^−1^) [50], Ni_0.32_Co_0.68_S_2_//AC (37.0 Wh kg^−1^ and 800 W kg^−1^) [51], NiCo_2_S_4_//mesoporous carbon (22.8 Wh kg^−1^ and 160.0 W kg^−1^) [30], NiCo_2_S_4_ hollow cages//AC (35.3 Wh kg^−1^ and 750 W kg^−1^) [35], NiCo_2_S_4_@Co(OH)_2_//AC (35.89 Wh kg^−1^ and 400 W kg^−1^) [52], NCS-graphene//AC (30.29 Wh kg^−1^ and 400 W kg^−1^) [53], Co_9_S_8_-NSA//AC (20.0 Wh kg^−1^ and 828.5 W kg^−1^) [54], and NiS//AC (31.0 Wh kg^−1^ and 900 W kg^−1^) [55]. For a practical application presentation, the CNS//AC HSC device was assembled into coin cells to demonstrate the durability in real-time applications. The images of serially accompanied two-coin cells and the “YU” LEDs pattern prior connecting with HSCs are shown in Figure 11a,b. Figure 11c,d validates the coin cells can light up “YU” pattern made up of 14 commercially available green LEDs connected in parallel. This superior HSC performance suggests that CNS//AC HSC device is suitable candidate for the next-generation energy storage applications.

## 4. Conclusions

In summary, we have demonstrated the facile one-step hydrothermal method to synthesize microsphere-like NCS and CNS electrode materials for efficient battery-type supercapacitor applications. The as-prepared both NCS and CNS bimetallic sulfide electrodes delivered excellent electrochemical properties such as specific capacity, rate capability, and long cycle stability in a three electrode-system. Surprisingly, the CNS electrode displayed a high specific capacity (430.1 C g^−1^ at a current density of 1 A g^−1^), better rate capability (58.14% capacity retention after 20 A g^−1^), and excellent long-term cycle stability (capacity retention of 84.7% after 10,000 cycles) than NCS electrode material. Afterward, a high-performance HSC device was fabricated with CNS as a positive electrode and AC as a negative electrode. The assembled CNS//AC HSC device delivered a high energy density of 41.98 Wh kg^−1^ at a power density of 800.04 W kg^−1^. Besides, our HSC device maintained 91.79% capacity retention even after 15,000 continuous GCD cycles. These electrochemical results suggest that the as-prepared NCS and CNS electrode materials are highly suitable for high-performance battery-type supercapacitors.

## Data Availability

The data presented in this study are available on request from the corresponding author.

## Supplementary Materials

The following supporting information can be downloaded at: https://www.mdpi.com/article/10.3390/nano12244435/s1, The details of materials and electrochemical characterization; Figure S1: Cyclic voltammetry curves of (a) NCS and (b) CNS electrodes in the non-Faradaic region at different scan rates from 50 to 250 mV s^−1^; Figure S2: Crystalline phase and surface morphology of the CNS electrode after 10000 cycles (a) XRD pattern and (b, c) FE-SEM images; Figure S3: Supercapacitor performance of negative electrode in the three-electrode system: (a) CV curves at various scan rates and (b) GCD curves at different current densities; Table S1: EIS fitted parameters for NCS and CNS electrodes; Table S2: Electrochemical performance comparison of the NCS and CNS electrode materials with sulfide-based electrode materials. References [17,22,25,38,46,47,52,56,57,58,59,60] are cited in the Supplementary Materials.
