# Supplementary material for: Rationally Designed Bimetallic Co–Ni Sulfide Microspheres as High-Performance Battery-Type Electrode for Hybrid Supercapacitors
Source: Nanomaterials (Basel). 2022 Dec 13;12(24):4435. doi: 10.3390/nano12244435 (PMC9784776; doi:10.3390/nano12244435)
Supplement: Supplementary file 1 [file nanomaterials-12-04435-s001.zip › nanomaterials-2093404-supplementary.pdf]

Supplementary Materials

Rationally Designed Bimetallic Co–Ni Sulfide Microspheres as high-Performance Battery-Type Electrode for Hybrid Supercapacitors

John Anthuvan Rajesh [1,†], Jong-Young Park [1], Ramu Manikandan [2,†] and Kwang-Soon Ahn [1,*]

[1] School of Chemical Engineering, Yeungnam University, Gyeongsan 712-749, Republic of Korea
[2] Department of Energy and Materials Engineering, Dongguk University-Seoul, Seoul 04620, Republic of Korea
* Correspondence: kstheory@ynu.ac.kr; Tel.: +82-53-810-2524; Fax: +82-53-810-4631
† These authors contributed equally to this work.

Experimental

Materials characterization

The crystalline phases of the as-synthesized NCS and CNS electrode materials were analyzed using powder X-ray diffraction (XRD, X'Pert-Pro, PANalytical). The surface chemical composition and valence states of the prepared materials were characterized by X-ray photoelectron spectroscopy (XPS, K-Alpha ESCA, Thermo Scientific). The surface morphologies, microstructure, crystallinity, and chemical composition of synthesized materials were analyzed using field-emission scanning electron microscopy (FE-SEM, Hitachi, S-4800), field-emission transmission electron microscopy (FE-TEM, Tecnai G2 F20), and energy dispersive X-ray analysis (EDX; Oxford Instruments).

Electrochemical characterization

Before performing electrochemical studies in a three-electrode system, the working electrodes were prepared by mixing of electroactive materials (NCS or CNS), acetylene black, and PVDF with a weight ratio of 85:10:5 in ethanol. The prepared mixture was sonicated for 30 min to obtain a homogeneous dispersion. Then, the as-prepared dispersion was drop-casted onto the Ni-foam substrate (area of 1 ×1 cm^2) twice on both sides and the resulting electrodes were dried in a vacuum oven at 70°C for overnight. Finally, the dried electrodes were pressed under a pressure of 10 MPa for 30 s. The loading masses of NCS and CNS electrodes were about 3.2 and 3.4 mg cm^{-2}, respectively.

The battery-type supercapacitor performances such as cyclic voltammetry (CV), galvanostatic charge-discharge (GCD), cyclic stability, and electrochemical impedance spectroscopy (EIS) were evaluated using three-electrode system on a WonATech, WBCS3000 electrochemical workstation. The as-fabricated NCS and CNS electrodes, Ag/AgCl, and a graphite rod were used as the working electrode, the reference electrode, and the counter electrode, respectively. An aqueous 3M KOH solution was used as the electrolyte. CV curves were performed in a potential window of –0.2 ~ 0.5 V (vs. Ag/AgCl) and GCD curves were recorded from –0.1 to 0.4 V (vs. Ag/AgCl), and EIS measurements were recorded in the frequency range from 100 kHz to 0.01 Hz with amplitude of 5 mV.

Further, the electrochemical properties of CNS microspheres electrode were evaluated by fabricating an HSC device. In this hybrid device, the activated carbon (AC) utilized as negative electrode and CNS microspheres used as positive electrode (labeled as CNS//AC). The negative electrode fabrication and their electrochemical performance study were reported in our previous work [1]. The mass ratio of the CNS microspheres (positive electrode, m^+, 3.4 mg) to AC (negative electrode, m^-, 22.6 mg) was determined using the charge balance relationship ($q^+ = q^-$) [2]. The electrochemical performance of the CNS//AC HSC device was tested in a 3M KOH. The CV curves of CNS//AC device was performed from 0 to 1.6 V at different scan rates (10 ~ 50 mV s^{-1}). The GCD analysis was

carried out at various current densities from 1 to 20 A g^{-1} within the optimized potential window of 0 ~ 1.6 V.

The specific capacity values of the NCS and CNS electrodes were calculated from the discharge curves according to the following equation.

$$Cs = (I\ \Delta t)/(m) \quad (1)$$

where Cs, I, m, and Δt, are the specific capacity (C g^{-1}), the discharge current (A), mass of the active material (g), and the discharge time (s), respectively.

The Coulombic efficiency (η) of the electrodes was calculated from the charge and discharge times using the following equation.

$$\eta = td/tc \times 100\% \quad (2)$$

where td and tc are the discharge and charge time, respectively.

The specific capacitance of the negative electrode is calculated according to the following equation:

$$Cs = (I\ \Delta t)/(m\ \Delta V) \quad (3)$$

where Cs, I, m, Δt, and ΔV are the specific capacitance (F g^{-1}), the discharge current (A), mass of the active material (g), the discharge time (s), and the operating window voltage, respectively.

The energy density and power density values of the CNS//AC HSC device were calculated using the equations 4 and 5.

$$E = C\ \Delta V^2/7.2 \quad (4)$$

$$P = 3600\ E/\Delta t \quad (5)$$

where E (Wh kg^{-1}) is the energy density, P (W kg^{-1}) is the power density, C (F g^{-1}) is the specific capacitance, Δt (s) is the discharge time, and ΔV (V) is the potential window.

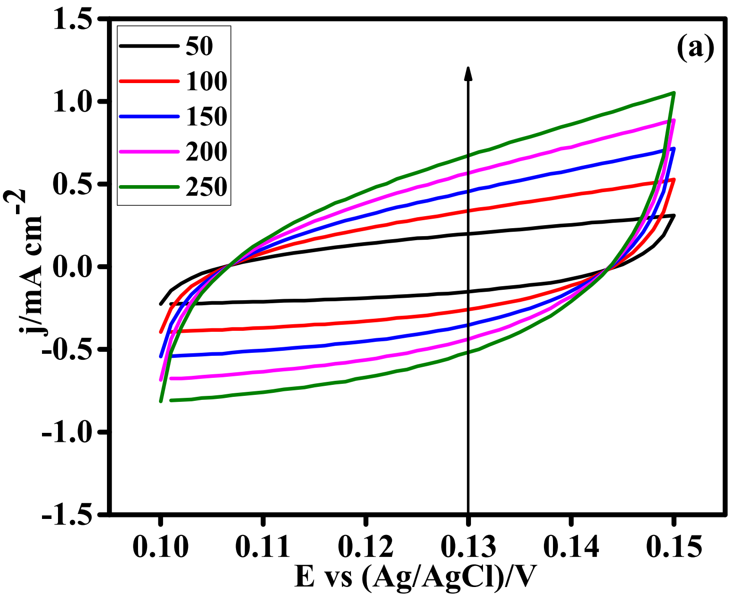

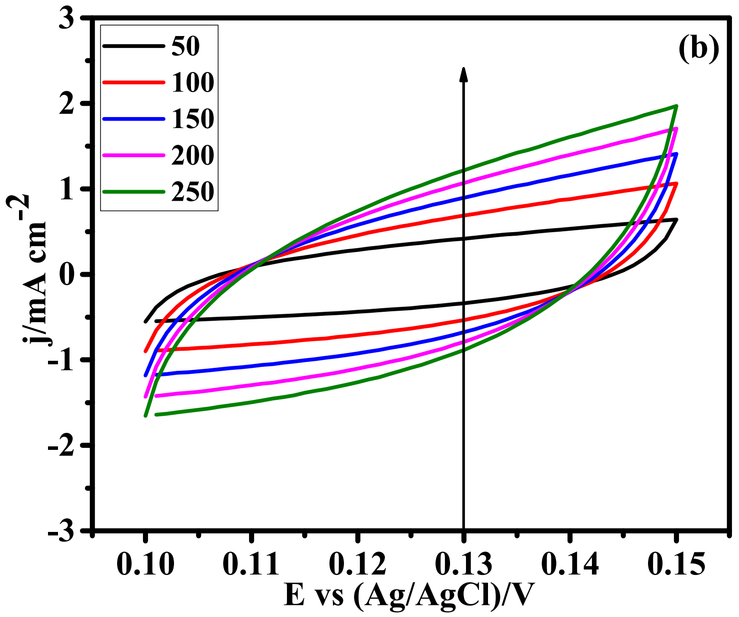

Figure S1. Cyclic voltammetry curves of (a) NCS and (b) CNS electrodes in the non-Faradaic region at different scan rates from 50 to 250 mV s^{-1}.

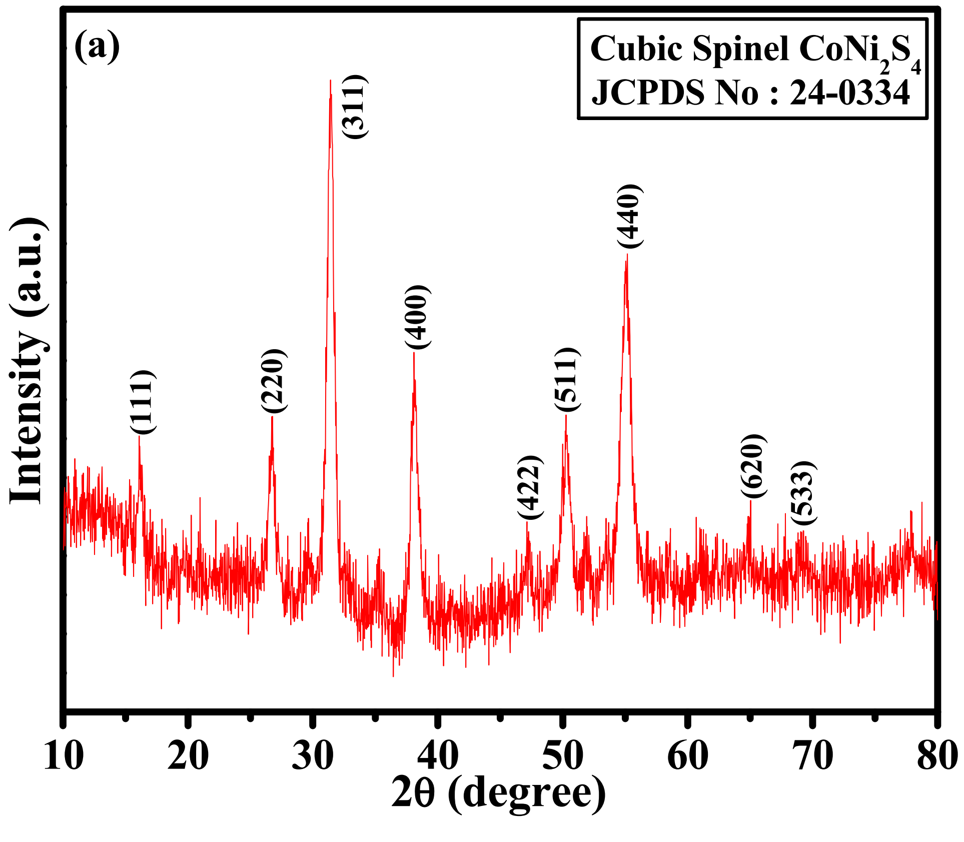

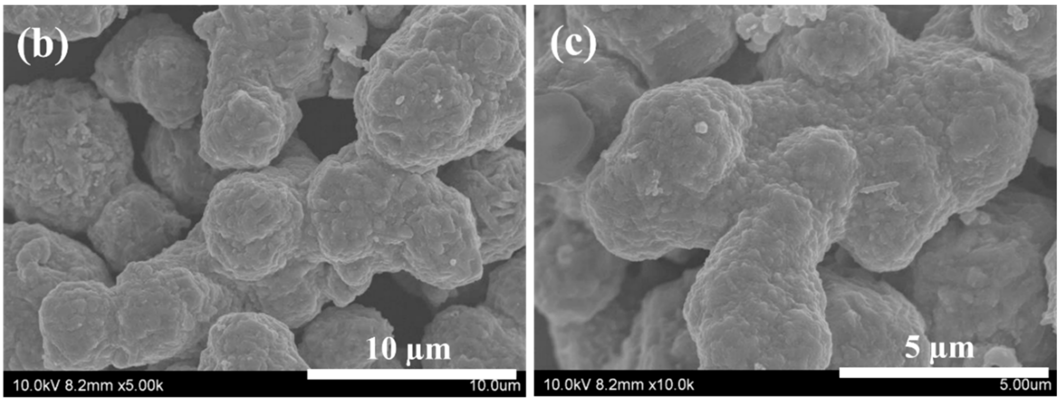

Figure S2. Crystalline phase and surface morphology of the CNS electrode after 10000 cycles. (a) XRD pattern and (b, c) FE-SEM images.

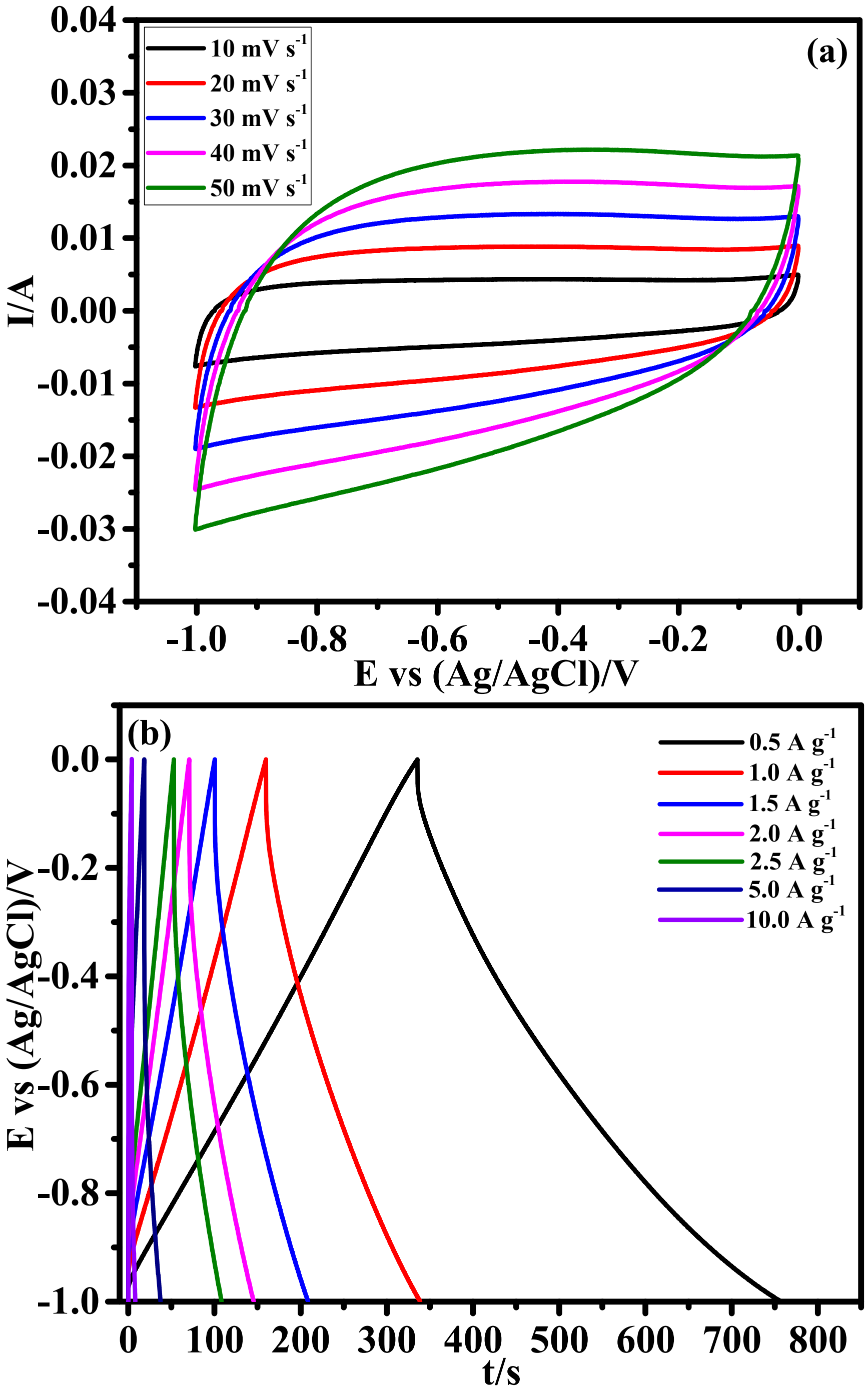

Figure S3. Supercapacitor performance of negative electrode in the three-electrode system: (a) CV curves at various scan rates and (b) GCD curves at different current densities.

Table S1. EIS fitted parameters for NCS and CNS electrodes.

Parameters	R_s (Ω cm^2)	R_{ct} (Ω cm^2)	W_R (Ω cm^2)	CPE1 (mF cm^{-2})	CPE2 (mF cm^{-2})
CNS microspheres	0.76	0.34	0.65	0.12	0.17
NCS microspheres	0.72	0.27	0.5	0.145	0.188

Table S2. Electrochemical performance comparison of the NCS and CNS electrode materials with sulfide-based electrode materials.

Electrode materials	Specific capacitance	Current density	Potential window	Electrolyte	Ref.

**CNS microspheres**	**860.2 F g^{-1}**	**1 A g^{-1}**	**-0.1-0.4 V**	**3 M KOH**	**This work**
**NCS microspheres**	**691.8 F g^{-1}**	**1 A g^{-1}**	**-0.1-0.4 V**	**3 M KOH**	**This work**
Needle-like $CoNi_2S_4$-MnOOH	803.5 F g^{-1}	0.5 A g^{-1}	0-0.32 V	6 M KOH	[3]
$NiCo_2S_4$ nanoplate	437.0 F g^{-1}	1 A g^{-1}	-0.1-0.4 V	3 M KOH	[4]
$CoNi_2S_4$ NPs/GO	755.0 F g^{-1}	4 A g^{-1}	0-0.4 V	3 M KOH	[5]
$CoNi_2S_4$ NPs	770.0 F g^{-1}	4 A g^{-1}	0-0.4 V	3 M KOH	[6]
$NiCo_2S_4$ nanoprism	895.0 F g^{-1}	1 A g^{-1}	0-0.5 V	2 M KOH	[7]
$(CoNi)O_xS_y$ nanoaggregates	592.0 F g^{-1}	0.5 A g^{-1}	-0.1-0.5 V	2 M NaOH	[8]
Urchin-like $NiCo_2S_4$	795.0 F g^{-1}	6 A g^{-1}	0-0.4 V	6 M KOH	[9]
Cubic-like $NiCo_2S_4$	450.0 F g^{-1}	6 A g^{-1}	0-0.4 V	6 M KOH	[9]
Co_9S_8 nanoparticles	734.0 F g^{-1}	1 A g^{-1}	0-0.5 V	6 M KOH	[10]
NiS nanosheets	845.0 F g^{-1}	1 A g^{-1}	0-0.5 V	2 M KOH	[11]
NiS/graphene NSs	845.0 F g^{-1}	1 A g^{-1}	0-0.4 V	6 M KOH	[12]

References

1. Rajesh, J.A.; Park, J.-Y.; Kang, S.-H.; Ahn, K.-S. Effect of molar concentration on the crystallite structures and electrochemical properties of cobalt fluoride hydroxide for hybrid supercapacitors. *Electrochim. Acta* **2022**, 414, 140203.
2. Li, R.; Wang, S.; Huang, Z.; Lu, F.; He, T. $NiCo_2S_4$@$Co(OH)_2$ core-shell nanotube arrays in situ grown on Ni foam for high performances asymmetric supercapacitors. *J. Power Sources* **2016**, 312, 156.
3. Qin, W.; Li, J.; Liu, X.; Zhou, N.; Wu, C.; Ding, M.; Jia, C. Formation of needle-like porous $CoNi_2S_4$-MnOOH for high performance hybrid supercapacitors with high energy density. *J. Colloid Interface Sci.* **2019**, 554, 125.
4. Pu, J.; Cui, F.; Chu, S.; Wang, T.; Sheng, E.; Wang, Z. Preparation and electrochemical characterization of hollow hexagonal $NiCo_2S_4$ nanoplates as pseudocapacitor materials. *ACS Sustainable Chem. Eng.* **2014**, 2, 809.
5. Du, W.; Wang, Z.; Zhu, Z.; Hu, S.; Zhu, X.; Shi, Y.; Pang, H.; Qian, X. Facile synthesis and superior electrochemical performances of $CoNi_2S_4$/graphene nanocomposite suitable for supercapacitor electrodes. *J. Mater. Chem. A* **2014**, 2, 9613.
6. Du, W.; Zhu, Z.; Wang, Y.; Liu, J.; Yang, W.; Qian, X.; Pang, X. One-step synthesis of $CoNi_2S_4$ nanoparticles for supercapacitor electrodes. *RSC Adv.* **2014**, 4, 6998.
7. Yu, L.; Zhang, L.; Wu, H.B.; Lou, X.W.D. Formation of $NixCo_{3-x}S_4$ hollow nanoprisms with enhanced pseudocapacitive properties. *Angew. Chem. Int. Ed.* **2014**, 53, 3711.
8. Liu, L. Nano-aggregates of cobalt nickel oxysulfide as a high-performance electrode material for supercapacitors. *Nanoscale* **2013**, 5, 11615.
9. Zhang, Y.; Ma, M.; Yang, J.; Sun, C.; Su, H.; Huang, W.; Dong, X. Shape-controlled synthesis of NiCo2S4 and their charge storage characteristics in supercapacitors. *Nanoscale* **2014**, 6, 9824.
10. Zhang, S.; Li, D.; Chen, S.; Yang, X.; Zhao, X.; Zhao, Q.; Komarnenid, S.; Yang, D. Highly stable supercapacitors with MOF-derived Co_9S_8/carbon electrodes for high-rate electrochemical energy storage. *J. Mater. Chem. A* **2017**, 5, 12453.
11. Yu, L.; Yang, B.; Liu, Q.; Liu, J.; Wang, X.; Song, D.; Wang, J.; Jing, X. Interconnected NiS nanosheets supported by nickel foam: Soaking fabrication and supercapacitors application. *J. Electroanal. Chem.* **2015**, 739, 156.
12. Li, Y.; Ye, K.; Cheng, K.; Yin, J.; Cao, D.; Wang, G. Electrodeposition of nickel sulfide on graphene-covered make-up cotton as a flexible electrode material for high-performance supercapacitors. *J. Power Sources* **2015**, 274, 943.
